# Microfollicular adenoma of ectopic thyroid gland masquerading as salivary gland tumor – a diagnostic and therapeutic challenge: a case report

**DOI:** 10.1186/1752-1947-8-270

**Published:** 2014-08-07

**Authors:** Sanjay D Deshmukh, Siddhi G Sinai Khandeparkar, Harveen K Gulati, Chetana S Naik

**Affiliations:** 1 Department of Pathology, Smt. Kashibai Navale Medical College & General Hospital, Narhe, Pune, Maharashtra, India; 2 Department of ENT, Smt. Kashibai Navale Medical College & General Hospital, Narhe, Pune, Maharashtra, India

**Keywords:** Ectopic thyroid, Follicular adenoma, Sublingual

## Abstract

**Introduction:**

Ectopic thyroid tissue may appear in any location along the trajectory of the thyroglossal duct from the foramen cecum to the mediastinum. Rarely, there is incomplete descent of the gland where the final resting point may be high resulting in sublingual ectopic thyroid tissue. Ectopic thyroid tissue carries a low risk of malignancy. Most recently reported neoplasms in ectopic thyroid tissue have been papillary carcinoma of thyroid. Individual case reports of clear cell type of follicular adenoma within the ectopic thyroid tissue have been described in the literature.

**Case presentation:**

We present a rare case of microfollicular follicular adenoma in an ectopic sublingual thyroid tissue presenting as submental swelling in a euthyroid 24-year-old Dravidian woman.

**Conclusion:**

Findings in this case emphasize that when confronted with a submental/sublingual mass lesion, the evaluation of thyroid function tests and ultrasonography of the neck should be included in a pre-operative workup.

## Introduction

Ectopic thyroid tissue (ETT) may appear in any location along the trajectory of the thyroglossal duct from the foramen cecum to the mediastinum. During the third to fourth week of gestation, the thyroid gland develops as an epithelial proliferation from the median plate of the floor of the pharyngeal gut. In approximately the seventh week of fetal life, the thyroid gland descends from the foramen caecum (located between the posterior third and anterior two thirds of the tongue) to its final location anterior to the pretrachea and larynx. Therefore, discrepancy in the descent of the thyroid gland may lead to ETT between the first and last positions of this gland. About 90% of all ETT are found in these regions, whereas about 10% have been reported in other anatomical locations [1,2]. ETT carries a low risk of malignancy [2]. Here we present a rare case of microfollicular follicular adenoma in an ectopic sublingual thyroid tissue presenting as submental swelling in a euthyroid 24-year-old woman.

## Case presentation

**Figure 1:**
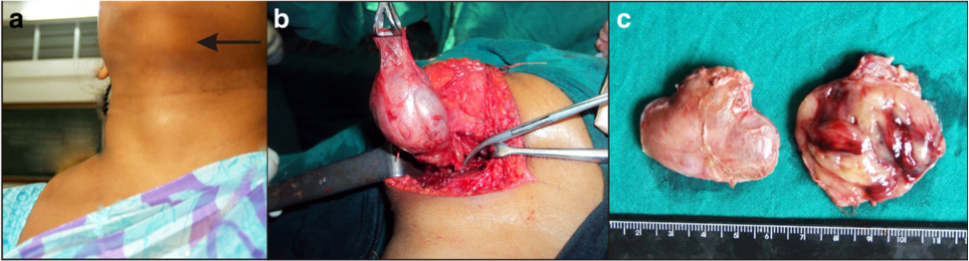
**Photographs of the patient during examination and surgery, and the removed tumor. (a)** Clinical photograph showing a swelling in anterolateral aspect of left submental region **(b)** Intraoperative photograph showing an encapsulated tumor. **(c)** Gross photomicrograph showing a single globular tissue. Cut section showing an encapsulated tumor with a homogeneous brownish area.

**Figure 2:**
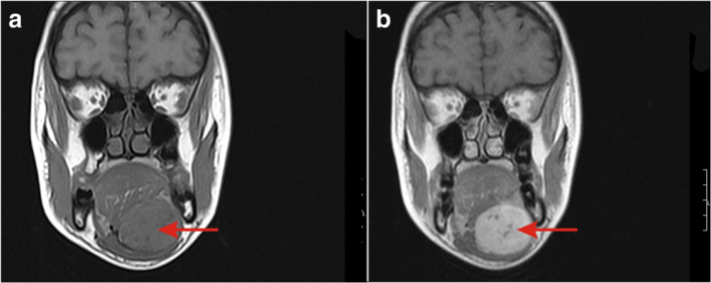
**Magnetic resonance images of the patient.** The image shows a well-defined contrast-enhanced round to oval mass (arrows) in the left sublingual region appearing isointense on T1-weighted images **(a)** and hyperintense on T2-weighted images **(b)**.

A 24-year-old Dravidian woman presented with swelling in her left submental region of 10 months’ duration which was gradually increasing in size. There was no history of pain, fever or odynophagia. Apart from a full-term delivery by Caesarean section 2.5 years earlier, there was no other significant past history. On examination, she was averagely built with a pulse rate of 78/minute and a blood pressure of 100/70mmHg. On local examination, a swelling was seen in her left submental region measuring 4.0×3.0cm (Figure 1a). The swelling was mobile and non-tender. There were no lymph nodes palpable in her neck. Magnetic resonance imaging (MRI) showed a well-defined contrast-enhanced round to oval mass in her left sublingual region appearing isointense on T1-weighted images, and hyperintense on T2W and short tau inversion recovery images (Figure 2). The lesion was seen to cross the midline and displaced her tongue superiorly and to the right. The imaging findings suggested salivary gland tumor probably arising from minor salivary gland. Fine-needle aspiration cytology (FNAC) revealed paucicellular smears showing few epithelial cells in sheets and in a vague glandular pattern. There was no evidence of malignancy in the smears examined. With a presumptive diagnosis of benign salivary gland neoplasm, she was taken up for excision of the swelling with conventional incision for submandibular gland excision.

**Figure 3:**
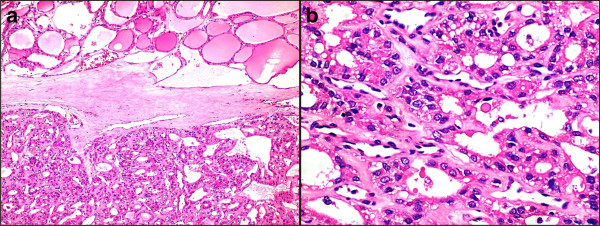
**Histology of the encapsulated tumor. (a)** Photomicrograph showing an encapsulated tumor composed of cells arranged in microfollicular, glandular and trabecular patterns (hematoxylin and eosin; 100×). **(b)** High power photomicrograph showing the microfollicles containing inspissated colloid resembling hyaline globules and separated by eosinophilic extracellular hyaline material. (hematoxylin and eosin; 100×).

**Figure 4:**
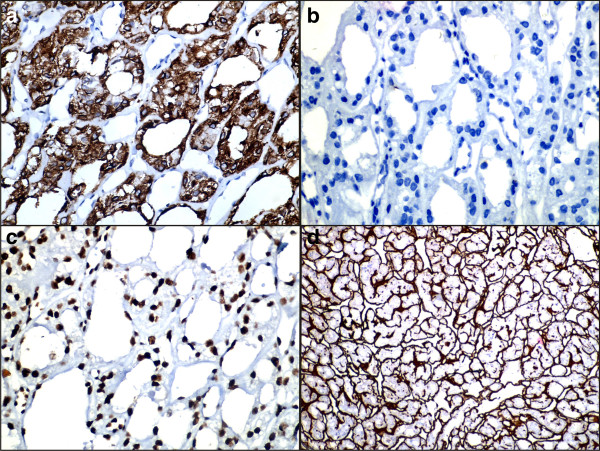
**Composite photomicrograph. (a)** Cytoplasmic and membrane positivity for cytokeratin 7 immunostain (400×); **(b)** negativity for cytokeratin 20 immunostain (400×); **(c)** nuclear positivity for retinoblastoma immunostain (400×); and **(d)** extracellular positivity for collagen type IV immunostain (400×).

Intraoperatively, an encapsulated tumor during excision appeared to be attached to the upper border of her hyoid bone (Figure 1b). It was excised and submitted for surgical pathology examination. Grossly, we received a single globular tissue measuring 4.0×4.0×4.0cm. Cut section revealed an encapsulated tumor measuring 4.0×4.0×3.7cm showing a homogeneous surface with areas of brownish discoloration. On the periphery of the nodule was seen a roughened area (Figure 1c). Sections examined showed an encapsulated tumor composed of cells arranged in microfollicular, glandular and trabecular patterns. The cells were cuboidal to columnar and showed a moderate amount of eosinophilic cytoplasm and central round to oval nuclei. The lumen contained inspissated colloid resembling hyaline globules. The intervening areas showed abundant eosinophilic extracellular hyaline material along with ectatic blood vessels. No capsular/vascular invasion was noted. The surrounding adjacent tissue showed non-neoplastic normal-looking thyroid follicles containing abundant colloid (Figure 3). There was no evidence of nuclear pseudo-inclusions, ground grass nuclei or nuclear grooves. With the above findings, a diagnosis of microfollicular adenoma within ectopic thyroid gland was offered. Immunohistochemistry corroborated the findings on microscopy. The cells showed cytoplasmic and membrane positivity for pan cytokeratin (CK) and CK7 (Figure 4a) and negativity for CK20 (Figure 4b), epithelial membrane antigen , galactin-3 as well as CK 19. The cells also showed nuclear positivity for retinoblastoma (Figure 4c). The hyaline stroma was highlighted with collagen type IV immunostaining (Figure 4d). Subsequently, an imaging and thyroid scan for localization of normal thyroid was advised along with postoperative triiodothyronine (T_3_), thyroxine (T_4_) and thyroid-stimulating hormone (TSH) monitoring. A postoperative thyroid scan and ultrasound examination of her neck showed nonvisualization of orthotopic thyroid gland in her anterior neck region. Thyroid hormone levels done after 10 days of excision revealed T_3_ level of 1.17pg/mL; T_4_ level of < 0.40ng/dL and TSH level of >100μIU/mL. She was advised thyroxine replacement therapy and was euthyroid after 6 months of follow up.

## Discussion

ETT is a rare developmental abnormality involving aberrant embryogenesis of the thyroid gland during its passage from the floor of the primitive foregut to its final pretracheal position. ETT is rare, with a reported incidence of 1 in 300,000. The ectopic thyroid was described for the first time, in 1869, by Hickman in a newborn [3].

Most commonly, the gland completely fails to descend and presents as a lingual thyroid. In 70% of the cases this is the sole thyroid tissue with no orthotopic thyroid gland. Rarely, there is incomplete descent of the gland where the final resting point may be high resulting in sublingual ETT. The sublingual ETT may be suprahyoid, infrahyoid or at the level of the hyoid bone [4].

When the thyroid gland is located in its normal position in the lower neck, fragments of the thyroid tissue representing ectopic thyroid may still be found anywhere along its course. Cases of ETT have also been documented in the submandibular region, trachea, esophagus or paraesophageal region, heart, aorta and superior mediastinum. A variety of unexpected sites have also been reported including lung, duodenum, gall bladder, porta hepatis, pancreas, small intestinal mesentery, adrenal gland, parotid gland, sella turcica and skin [5].

Genetic research has shown that the gene transcription factors *TITF1* (*NKX2-1*), *FOXE1* (*TITF2*) and *PAX8* are essential for thyroid morphogenesis and differentiation. Mutation in these genes may be involved in abnormal migration of the thyroid. It has also been shown in gene-targeting experiments that *FOXE1* is required for thyroid migration and that mice homozygous for *FOXE1* mutations show a sublingual thyroid [5].

ETT in the submental region is relatively rarely documented and is usually associated with a thyroglossal duct cyst [6]. Here we encountered a rare case of sublingual microfollicular adenoma presenting as submental swelling not associated with thyroglossal duct cyst. In 70% of ETT, the gland is not found in an orthotopic position. An asymptomatic mass lesion is the usual presentation of ectopic lingual thyroid, but obstructive symptoms, hypothyroidism and very rarely hyperthyroidism have also been documented. Sublingual ectopic thyroid commonly presents as an anterior neck mass above, below or at the level of the hyoid bone. It is usually painless, gradually increasing in size, and may move with swallowing. Characteristically, the mass has smooth margins and is soft in consistency, mobile and non-tender [1,7].

Of patients with ectopic lingual thyroid without a co-existing eutopic thyroid tissue, 70% develop subclinical hypothyroidism [1,7]. It is worth mentioning that in our case, our patient was euthyroid in spite of absence of orthotopic thyroid. In lingual ectopic thyroid, the swelling is usually located at or quite near the mucosal surface at the base of the tongue. Less often, they are located sublingually deeper in the musculature as in the present case [7]. ETT in the lingual, sublingual or laryngeal region may also present with dysphagia, dysphonia, and dyspnea often necessitating surgical removal [7].

ETT can be subject to the same pathological processes as normal eutopic thyroid tissue such as inflammation, hyperplasia, and rarely neoplastic transformation. The appearance of such tissue in rare locations often leads to diagnostic and therapeutic dilemmas.

The differential diagnosis includes thyroglossal duct cyst, epidermal cyst, lymphadenopathy, lipoma, lymphangioma, sebaceous cyst, cystic hygroma, dermoid cyst, midline branchial cyst and neoplasms [8].

ETT carries a low risk of malignancy (<1%). In older case studies follicular carcinomas are more prevalent. However, most recently reported neoplasms in ETT have been papillary carcinoma of thyroid owing to increased recognition of its follicular variant. Such is the case with sublingual ETT presenting as submental swelling [9]. Individual case reports of clear cell type of follicular adenoma within the ETT have been described in the literature [10]. However, our case is unique and showed histology with microfollicular pattern separated by hyalinizing stroma. Distinguishing follicular neoplasia in a sublingual thyroid is challenging given the common intermingling of ectopic tissue with skeletal muscle. The diagnosis of follicular carcinoma requires vascular invasion and unequivocal infiltration with desmoplastic response. In the present case the tumor was well encapsulated and did not show vascular invasion [11].

It is particularly significant to have ETT as one of the differential diagnoses of swellings presenting in the neck as inadvertent removal of ectopic thyroid gland leading to significant hypothyroidism has been reported in the literature [12]. Our experience with the present case is similar. To avert this crisis, routine preoperative identification of the normal thyroid gland by an ultrasound is advocated in all cases. When the thyroid gland can be identified in the normal position, coexistent ectopic thyroid is seldom found. Thyroid scintigraphy is the best method in identifying all sites of functioning thyroid tissue, but routine thyroid scan is not necessary. It is justified in cases of ectopic thyroid and where a normally located thyroid gland cannot be detected. A thyroid function test is an essential test and should be included in the evaluation of all cases of sublingual mass lesion. A computed tomography scan and MRI may help in defining the extension and location of the ectopic thyroid gland. FNAC can also be done; however, highly vascular lingual thyroid may bleed [5].

Our experience with the present case highlights the rarity of the lesion and the significance of detailed clinical, radiological and histological analysis of a mass lesion in the sublingual/submental region to judiciously avoid removal of the ectopic thyroid gland.

## Conclusion

Findings in this case emphasize that when confronted with a submental/sublingual lesion, the evaluation of thyroid function tests and ultrasonography of the neck should be included in the pre-operative workup.

## Consent

Written informed consent was obtained from the patient for publication of this case report and accompanying images. A copy of the written consent is available for review by the Editor-in-Chief of this journal.

## Abbreviations

CK: Cytokeratin; ETT: Ectopic thyroid tissue; FNAC: Fine-needle aspiration cytology; MRI: Magnetic resonance imaging; T_3_: Triiodothyronine; T_4_: Thyroxine; TSH: Thyroid-stimulating hormone; W: Weighted.

## Competing interests

The authors’ declare that they have no competing interests.

## Authors’ contributions

SDD had a major role in establishing the diagnosis histologically. SGSK was a major contributor in writing the manuscript. HKG performed the histopathological examination of the specimen. CSN treated the patient surgically. All authors have read and approved the final manuscript.
